# Re-exploring Dental Education During the COVID-19 Era: A Look Through the Challenges Faced, Solutions, and Their Impact on Traditional Methods

**DOI:** 10.7759/cureus.67106

**Published:** 2024-08-18

**Authors:** Sayem A Mulla, Minakshi Bhattacharjee, Sandhya A Methal, Amit Patil, Sarita Shrivastava, Sarita Mane

**Affiliations:** 1 Dentistry, Bharati Vidyapeeth (Deemed to be University) Dental College and Hospital, Navi Mumbai, IND; 2 Microbiology, Bharati Vidyapeeth (Deemed to be University) Dental College and Hospital, Navi Mumbai, IND; 3 Physiology, Bharati Vidyapeeth (Deemed to be University) Dental College and Hospital, Navi Mumbai, IND; 4 Conservative Dentistry and Endodontics, Bharati Vidyapeeth (Deemed to be University) Dental college and Hospital, Navi Mumbai, IND; 5 Pathology, Mahatma Gandhi Missions (MGM) Medical College and Hospital, Navi Mumbai, IND

**Keywords:** e-learning, dentistry, dental education, pandemic, covid-19

## Abstract

December 2019 brought to the world one of the worst pandemic diseases to exist - COVID-19. The pandemic led to worldwide lockdowns and quarantine, affecting the entire cycle of the world. The education system was one of the severely affected systems. Dental education, which is a practice-based learning system, faced complications due to the pandemic. E-learning methods were adapted in order to restrict the spread of the virus. E-learning has its own advantages and disadvantages. Although dental education is not possible by virtual means, there exist some methodologies that could be incorporated into routine teaching methods. The mixture of traditional learning methods along with newer methods adopted during COVID-19 can prove to improve the understanding procedure. The main aim of this article is to highlight these hybrid methods and encourage their incorporation to enhance the learning experience.

## Introduction and background

December 2019 brought to the world one of its worst public health crises into being - COVID-19 [1]. Spreading predominantly by respiratory droplet (aerosol)/contact, the severe acute respiratory syndrome coronavirus 2 (SARS-CoV-2), a virus that causes the COVID-19 disease, was present abundantly in nasopharyngeal and salivary secretions of affected patients. By profession, dentists are largely exposed to saliva and other body fluids, thus putting them at a heightened risk of contracting and spreading the disease [2].

With time, the virus spread more and more rapidly all across the world, and by March 2020, the World Health Organization (WHO) announced the outbreak as a pandemic. The increased risk of infection transmission led to changes in the delivery of health care services [3]. People (including dental students) were directed to quarantine, i.e., stay at home. Quarantine, though essential, to some extent seemed a bane for the dental students and dental schools. Right from physical learning, and practical up to in-hospital training, all the things were affected.

## Review

Effect of the pandemic on dental education

As the pandemic progressed, the regulating authorities had to make an impetuous decision and close the educational institutes to curb the increasingly harmful effects of the virus. To not let the students feel left out and put their and the nation's future in the stack, the government directed educational institutes to adopt an e-learning system for continuing education for students across the country (incl. dental students). Online classes thus became a part and parcel of students' daily lives. However, this induced a reduction and total loss of physical practical and pre-clinical/clinical exposure for the students. The aforementioned is one of the major drawbacks of online education, especially for dental students.

Dentistry is a discipline where patient care is the primary goal, the students need to attend clinics and provide patient treatment while acquiring their competencies. The DCI-approved curriculum for a Bachelor of Dental Surgery (BDS) includes two years of extensive preclinical training followed by two years of in-depth clinical training, which is finally terminated by one year of rotatory internship in all the dental specialty subjects and licensure to practice [4]. Dental chairside training is far more complex and technique-sensitive than higher education, as senior dental students manage the patient's oral health under the supervision of clinical specialists [5].

Lockdown and quarantine, however, had an impact on everything mentioned above. COVID-19 had repercussions on the dental educational system at all levels. Around 931,345,922 students in 94 countries, or 54% of all enrolled students worldwide, were impacted by the temporary closure of the academic institutions [6]. A total of around 35% of individuals succumbed to anxiety whereas 37% of people fell prey to depression [7,8]. Aside from getting COVID-19, dental students' stress was mostly caused by tests, loss of manual dexterity abilities, professional development, and the risk of transferring the disease to family members and/or flatmates while working in clinical practice. Sometimes contracting COVID-19 was not their main focus [9,10].

Due to high aerosol production in routine dental work, the clinics were temporarily shut down owing to the high chances of infection spread. Students, thus suffered from a lack of clinical skills augmentation. They were forced to switch to digital methods such as online tutorials by college staff or videos available online to imagine and work on the skills. However, a hidden challenge came into the picture with the shift to online education. This was nothing else but the lack of familiarity with online tools and applications on both ends, i.e., the student as well as the teacher. They thus found it difficult and fearsome to operate these gadgets on a daily basis.

Effect of the pandemic on dental research

Most dental students are interested in conducting dental research during their tenure as students to learn research methodologies. In reaction to the COVID-19 epidemic, academic institutions have reduced research activity [11]. Obtaining human tissue samples, such as removed teeth for dental research, was becoming more difficult as the lockdown limited the dental clinic activities. Additional human resources, time, and money were required for laboratory research. COVID-19 has caused fragmentation of ongoing clinical investigations and community trials. Clinical investigations were terminated due to government lockdowns and directives. Furthermore, lockdowns, travel problems, and even COVID-19 infection prevented some recruited volunteers from participating in ongoing research. As a result, many researchers, particularly PhDs, and other early-career researchers could not complete their laboratory investigations on time and had to seek additional financing from the restricted monies available for dental research. In an Australian study, over 90% of the researchers noted delays in meeting project goals, and 65% indicated delays in obtaining additional financing [12].

Methods employed by dental schools for continued dental education

**Figure 1:**
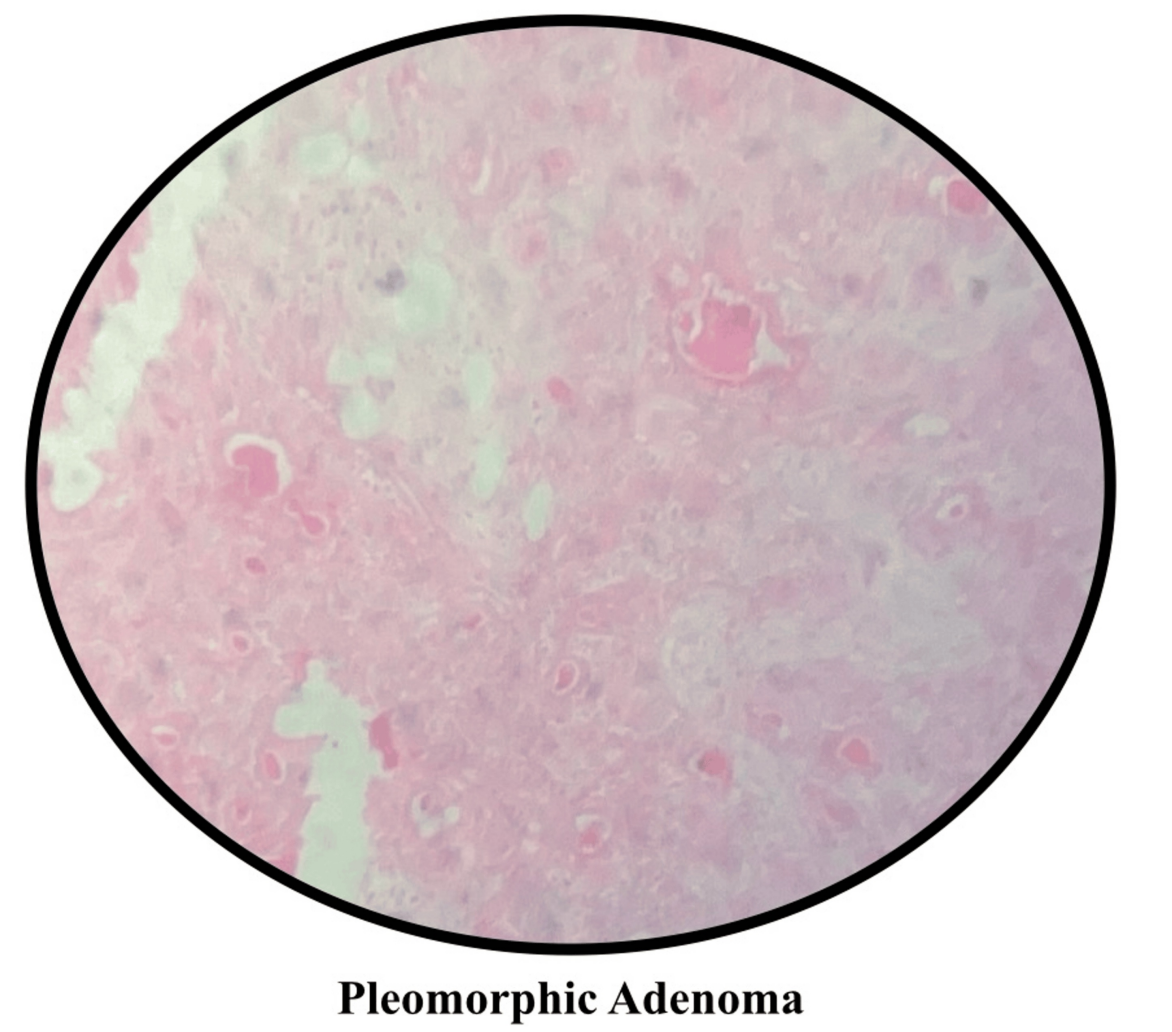
Digital histopathological slides were shown to students in order to explain the histopathology of various structures. Courtesy: Department of Pathology, Bharati Vidyapeeth (Deemed to be University) Dental College and Hospital, Navi Mumbai, MH, India.

**Figure 2:**
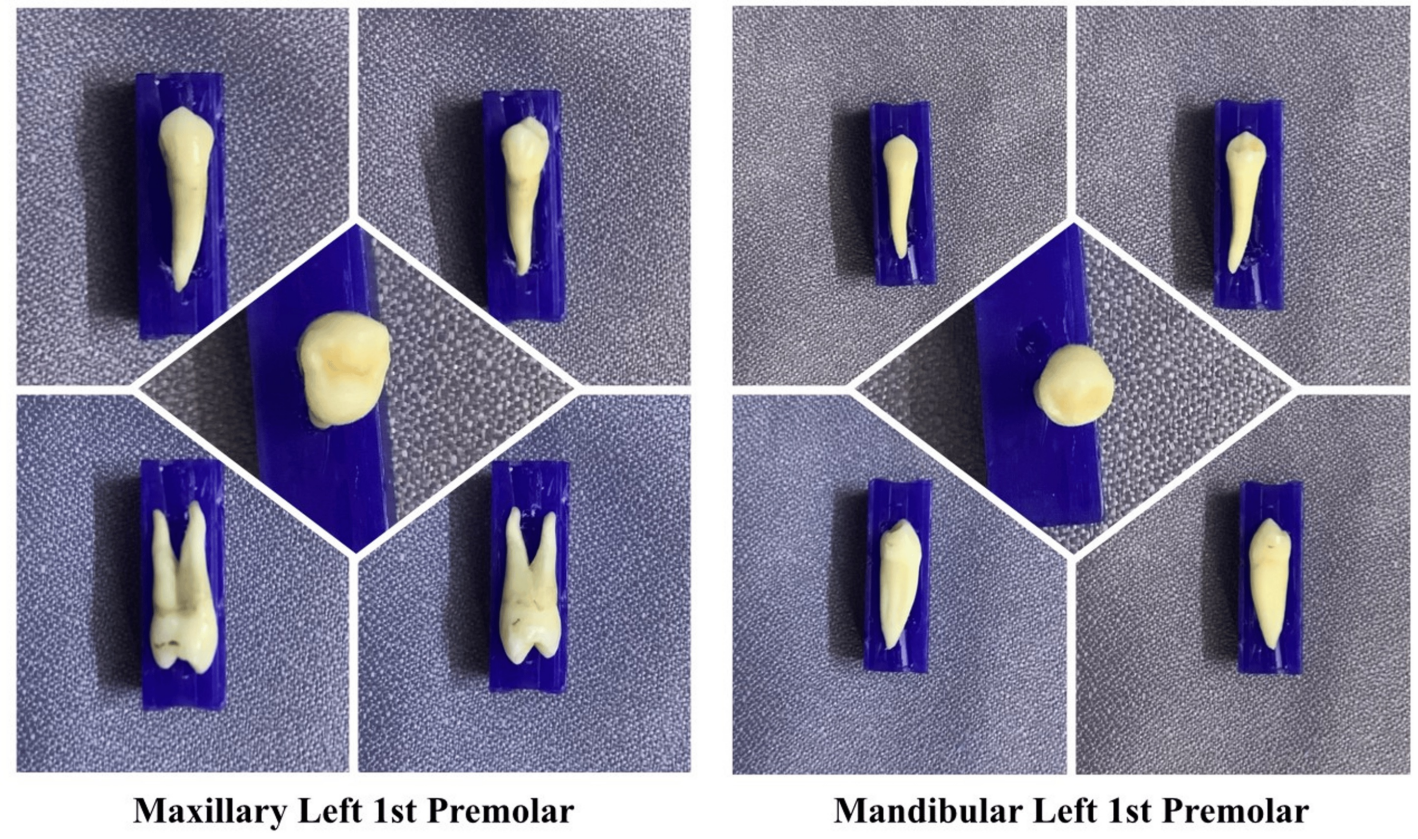
Tooth specimens were shown by putting them on display during carving demonstration classes in order to allow the students to have a better look and understanding of the specific tooth morphology. Courtesy: Sayem A Mulla.

Despite the lockdown, the Government of India encouraged dental schools to continue learning through virtual means. Various online meeting applications, such as Microsoft Teams, Google Meet, and Zoom, were used for theory lectures as well as practical/preclinical/clinical demonstrations [13,14]. Online histological and pathological slides were shown (Figure 1). Pathologists also adopted whole-slide imaging to enable easy teaching-learning and for better visualization of slides [15]. Virtual cadaveric dissection classes were held. Online demonstrations were given, and practice sessions were kept for wax tooth carving (Figure 2). The pre-clinical departments emphasized practicing the procedures via the means of mannequins as well as AR and VR techniques. During the pandemic, augmented and virtual reality came out as a friend for many healthcare educators and educatees [16-18].

Apart from this, the clinical departments, to continue their practical classes and sessions and clinical postings, started using dental models, typodont, and phantom heads with water inlets to simulate a patient's oral cavity. This helped conduct clinical/practical classes and allowed the educators to take the appropriate assessment without much difficulty. They assisted the educators in demonstrating various vital procedures simplified, whereas the students had the flexibility to look at multiple essential norms and practices one ideally would follow and impart in practical physical classes/clinical postings. Conferences, seminars, various scientific fests, and competitions became available online, providing the flexibility to the participants to participate in maximum numbers at the ease of their homes. Online conferences posed an added advantage of having prestigious speakers from all across the globe available to many audiences and listeners. Table 1 lists some of the activities and methods employed by dental schools to keep the learning environment as normal as possible.

**Table 1:** Activities conducted by educators to keep online dental learning fun, innovative, and insightful.

Activities conducted
Virtual histological/histopathological slides to better look at different physiological and pathological changes occurring in the body.
Online dissection classes.
Use of typhodonts and mannequins.
Polls and end-of-course quizzes kept the students engaged.
Online paper, poster, essay, art, and quiz competitions integrated knowledge with fun.
Online seminars and case presentations kept the students up-to-date with their clinical knowledge.
Virtual fests and prestige guest lectures from faculties across the globe kept the creativity bar set.
Online assessment using Google Forms to assess the student's gain during the pandemic.

Difficulties faced during COVID-19 in dental education

Even though the use of virtual and augmented reality has grown in popularity during the COVID-19 era, they do not appear to be able to compete with or replace direct in-person or hands-on training. Faculty faced a variety of challenges, including difficulty tracking the extent of knowledge students gained during the lecture, assessment issues, and difficulties in making concepts easier to understand. Students found it difficult to focus on lectures while also maintaining a healthy screen time balance. Dental education, which is heavily based on skill acquisition and assessment, suffered the most [14]. A significant reduction in the patient in-flow was seen during the COVID-19 period [19-21]. This directly affected the hands-on clinical skills of the dental students.

Disparities between students able to access IT technologies and those living in rural places where technological facilities are not available were also one of the major concerns [22]. Disruptions in standard academic calendars, the admission of new students, and the postponement of graduation ceremonies were among the pandemic's negative effects on routine educational activities. The pandemic is notoriously linked with a high incidence of mental trauma in both students as well as faculties along with unsurety of their future regarding academic growth, clinical excellence, and financial stability [20,23].

Advantages of methods employed during COVID-19 in dental education and their incorporation with traditional educational methods

The pandemic, though, proved to be a challenging time for the entire world; it did leave some useful methods that can be integrated with traditional learning methods. These hybrid methods can benefit both; the faculties and students and hold a promising grip in enhancing the quality of educational systems. It has allowed the opportunity to dental schools to leverage the technologies in ways that can enhance dental learning [24].

Using AR-VR as an adjunct in clinical training for better hands-on skills [16-18]. The use of virtual learning tools saves time for both students and teachers while also accommodating a larger group in a single sitting [25]. Sharing online PowerPoint presentations in addition to the traditional class-based lectures helps in allowing the students to get accommodated to the topic in an easier and more effective way. Virtual dissection and clinical work demonstrations along with traditional hands-on training, and the use of online assessment tools periodically in conjunction with traditional pen-and-paper assessment can help instill knowledge in the students in a much better way. However, these tools should be aimed toward being an adjuvant because they cannot replace the traditional teaching-learning methods [16-18,26].

Cone-beam computed tomography (CBCT) has been a blessing in the dental field. The availability of a 3D picture boosts it as a potential savior in the dental field. The use of this is not only limited to disease management but also in procuring diagnostic models. Once an image is formed it can be used in conjugation with 3D scanning and printing software to produce study models. This proved to be an excellent boon during the COVID-19 period when older records were scanned and diagnostic and/or study models were printed so that students could practice routine clinical work on a more accurate and real-life scenario when compared to pre-formed, ideal models. Additionally, this procedure has been used in the training of endodontic procedures, preclinical prosthodontics practicals as well as in bone grafting sessions for the reconstructive of the bone [27,28].

Due to the high contamination and spread rate, special cares were taken by the dental hospitals to reduce this risk. This was done by scheduling appointments, maintaining social distance, etc. This allowed the students to get accustomed to managing patients later in the practice in a sterile and healthy environment. The pandemic also threw light on how it affected the mental well-being of millions of people including dental professionals. The pandemic taught us that it is of utmost importance to incorporate a psychological cell in every dental school to help the students as well as the faculties [29].

Takeaway message

Any dental student's goal is to be able to provide the finest treatment possible; yet the decreasing number of patients seen in the clinic may jeopardize their education. As a result, dental education will most likely need to alter its curriculum in order to retain the level of practical skills obtained before and after graduation. Dental students will be able to study in a variety of ways other than treating patients by adopting new technological devices, equipment, haptic systems, virtual reality, simulation-based instruction, and 3D printers. Dental education should recognize that the times are changing, and the field of translational research involving dentistry, medical professionals, and engineers will undoubtedly present future opportunities to incorporate new teaching modalities. The COVID-19 compelled society to modify and adapt several daily operations. A pandemic that caused a global lockdown may have contributed to the interplay of science and teaching approaches for a new age in dentistry [30].

## Conclusions

Thus, we can conclude that, while the pandemic hit our country hard, it did not halt learning procedures in various dental schools across India or even the world. It certainly changed, slowed, and revolutionized traditional didactic methods, but dental schools found a way out and kept their pace. The virus may have caused some uncertainty at first, but as the situation became more common, the authorities worked hard to resolve the crisis. People are now immunized, and dental schools have begun to operate on a regular basis, integrating traditional methods with newer and better approaches to provide the best exposure to every student and produce better clinicians in the future.
